# The bacterial *yjdF* riboswitch regulates translation through its tRNA-like fold

**DOI:** 10.1016/j.jbc.2022.101934

**Published:** 2022-04-12

**Authors:** Robert J. Trachman, Luiz F.M. Passalacqua, Adrian R. Ferré-D’Amaré

**Affiliations:** Biochemistry and Biophysics Center, National Heart, Lung, and Blood Institute, Bethesda, Maryland, USA

**Keywords:** tRNA, RNA structure, riboswitch, translation, DMS, dimethyl sulfate, NHLBI, National Heart, Lung, and Blood Institute, RBS, ribosome-binding site, SAXS, small-angle X-ray scattering, SEC, size-exclusion chromatography, SHAPE, selective 2′-hydroxyl acylation analyzed by primer extension, TLC, T-loop chimera, TYMV, turnip yellow mosaic virus, *V*_e_, elution volume

## Abstract

Unlike most riboswitches, which have one cognate effector, the bacterial *yjdF* riboswitch binds to diverse azaaromatic compounds, only a subset of which cause it to activate translation. We examined the *yjdF* aptamer domain by small-angle X-ray scattering and found that in the presence of activating ligands, the RNA adopts an overall shape similar to that of tRNA. Sequence analyses suggested that the *yjdF* aptamer is a homolog of tRNA^Lys^, and that two of the conserved loops of the riboswitch are equivalent to the D-loop and T-loop of tRNA, associating to form an elbow-like tertiary interaction. Chemical probing indicated that this association is promoted by activating ligands such as chelerythrine and harmine. In its native mRNA context, activator ligands stabilize the tRNA-like fold of the *yjdF* aptamer, outcompeting the attenuated state in which its T-loop base pairs to the Shine–Dalgarno element of the mRNA. Moreover, we demonstrate that the liganded aptamer itself activates translation, as authentic tRNAs, when grafted into mRNA, can potently activate translation. Taken together, our data demonstrate the ability of tRNA to function as a small-molecule responsive *cis* regulatory element.

The *yjdF* riboswitch binds chemically diverse azaaromatic compounds, inducing gene expression in response to a subset of these chemicals (Fig. 1*A*). The ability of this bacterial mRNA element to respond to multiple compounds makes it unusual among riboswitches (1, 2). The *yjdF* riboswitch was discovered through bioinformatic analyses, and experimental RNA folding and gene expression studies demonstrated that upon binding activator azaaromatics, it adopts an alternative base-pairing arrangement to regulate translation in *cis* (3, 4)*.* Notably, all its activators have antimicrobial activity, making this riboswitch functionally analogous to multidrug binding proteins (5, 6, 7).

**Figure 1:**
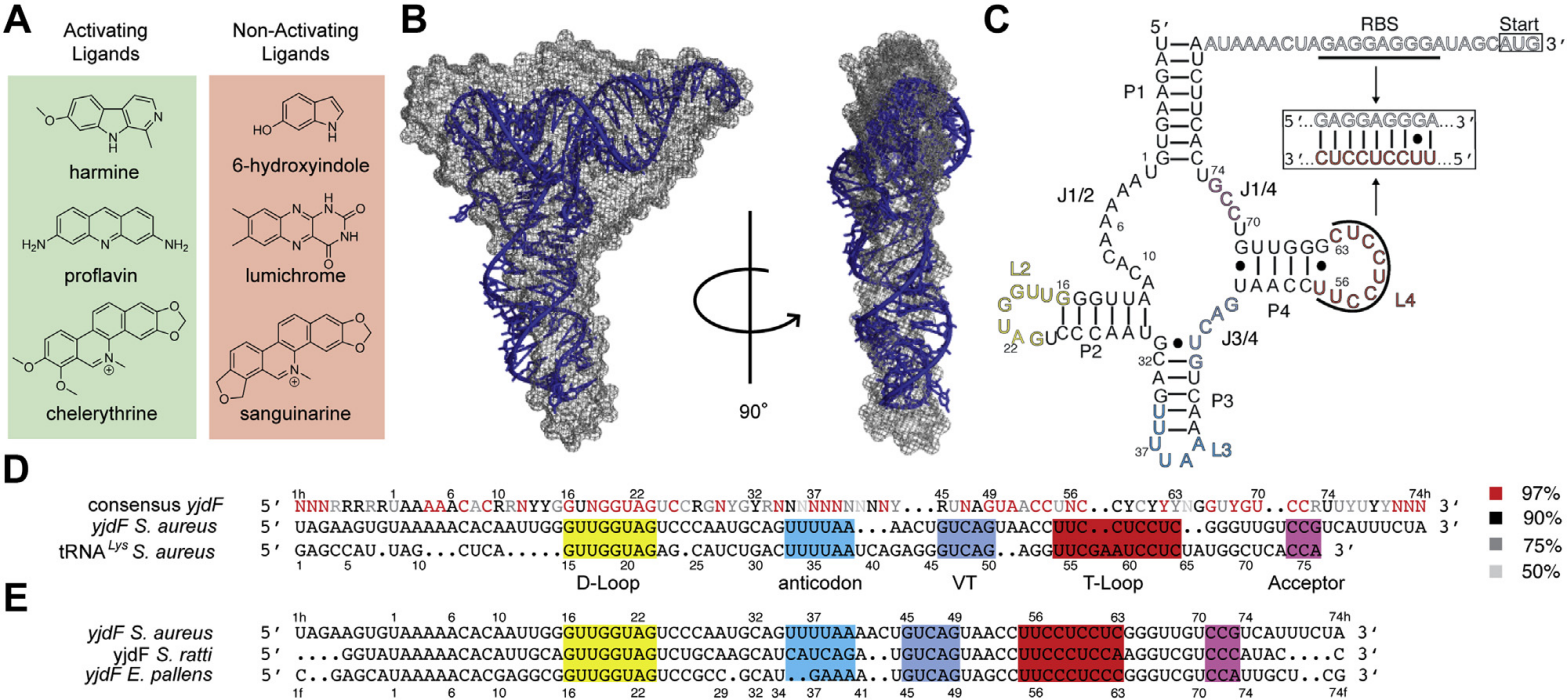
**Structural analysis and sequence comparison of the *yjdF* riboswitch.***A*, subset of ligands demonstrated to induce folding of the *yjdF* riboswitch and have an activating (*green box*) or no effect (*pink box*) on translation. *B*, molecular envelope generated from SAXS of the *Staphylococcus aureus yjdF* riboswitch, bound to chelerythrine, superimposed with the X-ray crystal structure of yeast tRNA^Phe^ (Protein Data Bank ID: 1EHZ) (28). *C*, secondary structure prediction of the *S. aureus yjdF* riboswitch aptamer domain (*black and colored lettering*). Paired elements of the four-helix junction are labeled P1–P4, with corresponding loops labeled L2–L4 and junctions labeled J1/2–J1/4. Expression platform containing the Shine–Dalgarno ribosome-binding site (RBS) and the AUG start codon (*boxed*) are shown in *gray lettering*. A predicted H-type pseudoknot, formed in the unbound “off-state,” is depicted in the *boxed subpanel*. *D*, sequence alignment of the consensus *yjdF* riboswitch sequence, *S. aureus yjdF* sequence, and *S. aureus* tRNA^Lys^. Consensus sequence is colored according to the percentage of occurrence (*right panel*) (3). Stem–loop regions from the predicted secondary structure model are colored according to (*C*). *E*, sequence alignment of three *yjdF* aptamer domains from *yjdF* orthologs. SAXS, small-angle X-ray scattering.

To investigate the mechanism by which this riboswitch recognizes and discriminates ligands, we characterized the ligand-binding (aptamer) domains of three *yjdF* riboswitch orthologs (*Staphylococcus aureus* [*S. aureus*], *Streptococcus ratti* [*S. ratti*], and *Enteroccocus pallens* [*E. pallens*]). Unexpectedly, the low-resolution molecular envelope of the RNA, derived from small-angle X-ray scattering (SAXS) experiments, revealed an overall shape similar to that of tRNA. Sequence alignments reveal conservation of loop regions, essential for forming tertiary structure, with that of tRNA^Lys^. Structural probing and mutagenesis show that the ligand-binding site of the riboswitch is located within an adenine-rich four-way junction, and that activating ligands preferentially stabilize a native fold that mimics tRNA shape. Indeed, the isolated *yjdF* riboswitch aptamer domain bound ribosomes and acts as a substrate in translation when bound to activating azaaromatics. The tRNA fold is versatile, having numerous biological functions beyond its canonical role in translation (8, 9, 10, 11, 12). Our studies now demonstrate that a tRNA-like element (defined here as an RNA with a four-way junction, containing tertiary structure that imparts an L-shaped fold) can fold selectively upon binding small molecules and control gene expression in *cis* when located upstream of the translation start site of an mRNA.

## Results

### The *yjdF* riboswitch is homologous to tRNA^Lys^

An RNA construct comprising the *S. aureus yjdF* aptamer domain was analyzed by SAXS, after purification by size-exclusion chromatography (SEC). *Ab initio* reconstruction (13) from the SAXS data produced an L-shaped molecular envelope reminiscent of tRNA. Superposition of the yeast tRNA^Phe^ (Fig. 1*B*) or the turnip yellow mosaic virus (TYMV) tRNA-like element X-ray crystal structures onto the envelope yields normalized spatial discrepancies (14) of 2.04 and 1.55, respectively. In comparison, superposition of the L-shaped SAXS envelopes of tRNA^Lys^ and the TYMV tRNA-like element (Fig. S1, *A*–*E*) with their respective crystal structures produces normalized spatial discrepancy values of 1.63 and 1.42.

Previous studies (3, 4) of the *yjdF* riboswitch did not report homology between this riboregulator and tRNA. The predicted secondary structure (Fig. S1, *F* and *G*) of the *S. aureus yjdF* aptamer domain can be redrawn to resemble a tRNA cloverleaf with the exception of an extended purine-rich J1/2 sequence element (Fig. 1*C*). Conventional sequence similarity searches (BLASTN and discontiguous BLAST (15)); for the *S. aureus yjdF* aptamer domain against a genomic and a tRNA database (tRNAScan-SE 2.0) (16) did not identify similarity to tRNAs or tRNA-like RNAs. However, we found strong sequence similarity between the highly conserved D-loop and T-loop of tRNA^Lys^ and tRNA^Phe^ within the *Streptococcus* genus and predicted loops of *Streptococcus yjdF* orthologs (Fig. 1*D*). The sequence of *S. aureus yjdF* L3 (although highly variable in orthologous riboswitches) is identical to a 6-nt sequence in the anticodon loop of *S. aureus* tRNA^Lys^ (Fig. 1*D*). In addition, a 5-nt sequence that encompasses a portion of the V-loop and T-stem in tRNA^Lys^ is conserved in 47% of 370 *yjdF* seed alignment representatives (17) (Fig. 1*E* and File S1). An alignment between tRNA^Lys^ and *S. aureus yjdF* demonstrates 62% sequence identity between nucleotides encompassing the D-loop to CCA tail (Fig. S1*H*). Together, these observations suggest an evolutionary relationship between *yjdF* and tRNA^Lys^.

### Ligand binding induces a tertiary interaction between L2 and L4

**Figure 2:**
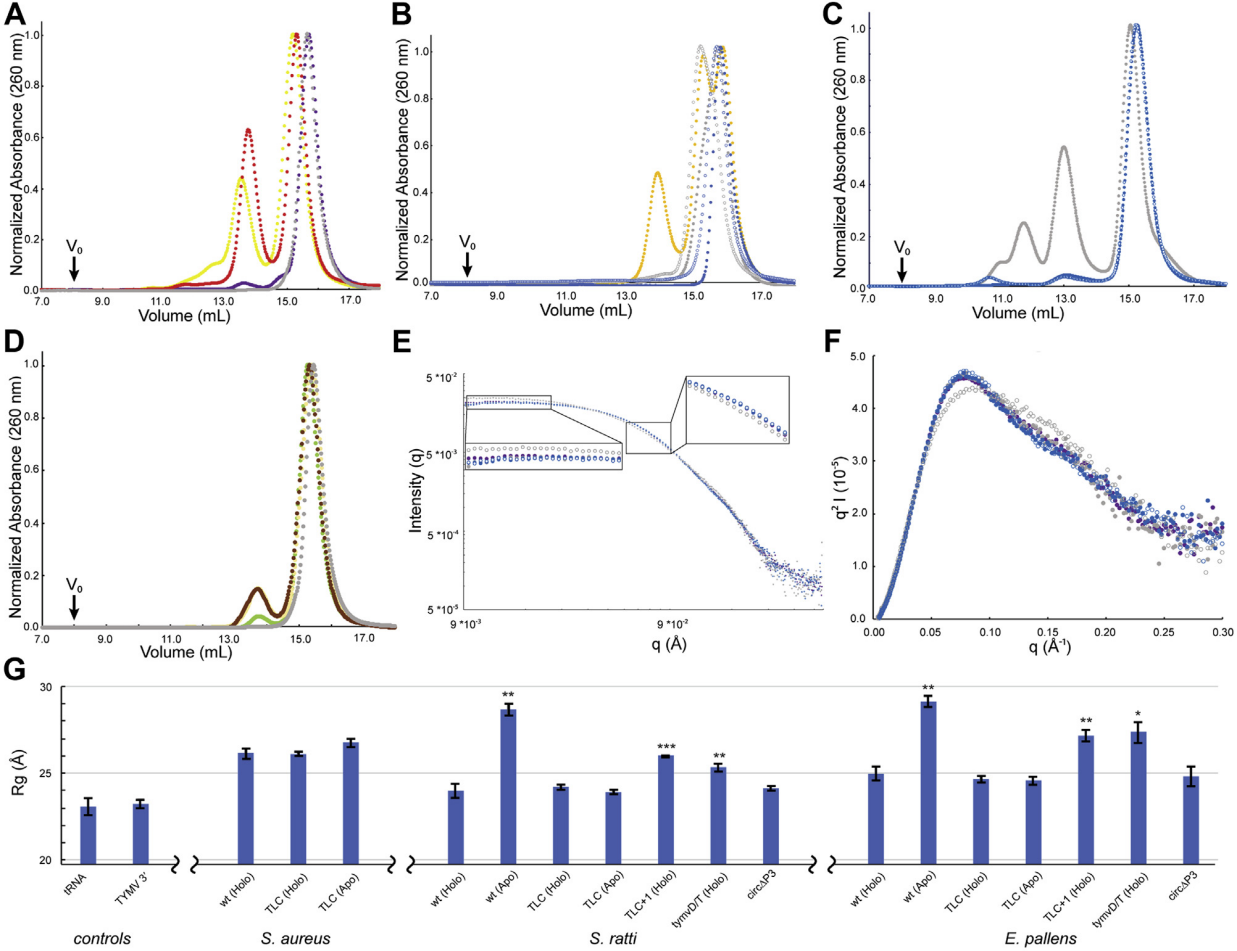
**Folding of the *yjdF* aptamer domain.** SEC and SAXS analyses of *yjdF* variants. Throughout, *closed circles* denote conditions in the presence of 60 nM chelerythrine, *open circles* denote conditions in the absence of ligand, and *gray circles* (*wt*). *A*, SEC of *Streptococcus ratti* circular permutants P2 (*red*), P3 (*purple*), and P4 (*yellow*). *B*, SEC of *Enteroccocus pallens* L4 mutants, TLC (*blue*), TLC+1 (*honey*). *C*, SEC of *Staphylococcus aureus* TLC mutant (*blue*). *E*, radially averaged SAXS profile of *S. ratti* wt, TLC mutant (*blue*), and circular permutant P3 (*purple*). *Subpanels* show magnified views of low-q and mid-q regions. *F*, Kratky plot of scattering profiles from (*E*). *G*, bar graph of the radius of gyration, determined from real space SAXS profiles, of tRNA^Lys^, TYMV 3′, and *yjdF* homologs and mutants. SAXS, small-angle X-ray scattering; SEC, size-exclusion chromatography; TLC, T-loop chimera.

We hypothesized that loops L2 and L4 would be structurally homologous to the D-loop and T-loop and associate, forming a tertiary interaction in the folded state. To test this, we constructed circular permutants by connecting the 5′ and 3′ ends of the riboswitch aptamer domain with a UUCG tetraloop and individually deleting loops L2, L3, or L4 (Table S1). SEC analysis of these permutants (circΔL2, circΔL3, and circΔL4, respectively) shows that deletion of L2 and L4 leads to aggregation (Figs. 2*A* and S2*A*) and smaller elution volume (*V*_e_) (Fig. 2*A*). CircΔL3 exhibits a modest increase in aggregation and reduced *V*_e_, suggesting that L3, which would be homologous to the anticodon loop, is not essential for folding.

We hypothesized that L4 may be required to form tertiary contacts, like those of the T-loop of tRNA, in response to binding small-molecule ligands. As a test, we mutated residues 55 to 63 of several *yjdF* orthologs (Fig. 1*E*) to the sequence of the T-loop (residues 54–64) of tRNA^Lys^ (Fig. 1*D*) to produce *yjdF*-T-loop chimeras (TLCs). Both *S. ratti yjdF* and *E. pallens yjdF* TLCs showed substantially reduced dependence on the activating ligand chelerythrine for folding (Figs. 2*B* and S2*B*). When L4 residues 58 to 61 were mutated to “GAAC” (L4mut), this resulted in increased aggregation and unfolding (Fig. 2*B*). Whereas the wt *S. aureus yjdF* aptamer domain is prone to aggregation under our experimental conditions, its TLC mutant exhibited reduced aggregation and an increase in *V*_e_ regardless of the presence of ligand (Fig. 2*C*). The improved folding of the mutant aptamer is consistent with ligand-dependent association of L2 and L4 in the native riboswitch.

To characterize the dimensions, folding, and flexibility of *yjdF* aptamer domain representatives and mutants, we performed SAXS on the subset of constructs that were monodisperse or maintained a monomeric population after SEC. The most substantial change in dimensions and flexibility arose from ligand binding (Fig. 2, *E*–*G*). X-ray scattering profiles of TLCs in both the presence and absence of ligand were identical to those of the folded wt (Figs. 2*E* and S2, *D*–*F*). The TLC mutants were also compact (as judged by Kratky analysis) in the absence of ligand (Figs. 2*F*, S2, *G* and *H*). However, the loop mutants tymvDT and L4mut differ in both flexibility (Fig. S2*G*) and compaction (Fig. 2*G*). Thus, restoration of an elbow-like pseudoknot is sufficient to stabilize a folded *yjdF* aptamer.

To determine if the reconstituted tRNA-like elbow interaction of TLC supports a native-like binding pocket, we sought to measure the response of *yjdF*, TLC, and a subset of mutants to binding of azaaromatic compounds. We chose to characterize the fluorescence change of ligands upon RNA binding, rather than calorimetry, because the latter can also respond to nonspecific interactions such as intercalation. A subset of azaaromatic compounds exhibit an altered fluorescence emission profile upon binding *yjdF*. Hyperchromic emission of 10-fold, 10-fold, and 1.1-fold results from *yjdF* binding to the ligands chelerythrine, sanguinarine, and harmine, respectively (Fig. S3, *A*–*C*). In addition, bathochromic emission shifts of 25 and 10 nm are observed for chelerythrine and harmine, respectively. Mutants that either maintain the wt L4 sequence (circΔP3) or impart a canonical tRNA^Lys^ elbow (TLC) elicit identical fluorescence emission spectra to the *yjdF* aptamer.

Our results demonstrate that formation of a tertiary interaction between L2 and L4, either by the wt or a chimeric mutant bearing sequence elements of tRNA^Lys^, is mutually inclusive to the conformation of the ligand-binding pocket of the riboswitch. *K*_*d*_s were measured for harmine binding to *yjdF*, circΔP3, and TLC using the fluorescence emission signal. Titration of *yjdF* (Fig. S3*D*) results in a distinctive biphasic curve. Both *K*_*d*_s and the curve shape for the *yjdF* mutants circΔP3 and TLC are nearly identical to those of wt (*K*_*d*_ = 230 ± 30 nM) and agree with the previously reported affinity for harmine (3).

### J1/2 forms a promiscuous ligand-binding pocket

To determine which riboswitch nucleotides comprise the ligand-binding pocket, we performed selective 2′-hydroxyl acylation analyzed by primer extension (SHAPE) and dimethyl sulfate (DMS; Sigma–Aldrich) chemical probing on the *S. ratti* wt and TLC mutant in the presence of various ligands (Fig. S3, *E*–*H*). The partially preorganized structure of these variants likely restricts the changes in nucleotide reactivity to residues directly involved in ligand binding and excludes those coupled to long-range tertiary interactions. Indeed, the SHAPE and DMS reactivities are similar between the wt and TLC variants and also between variants in the absence and presence of various ligands (Fig. S3*E*). However, two ligands that differ by a single methyl group, chelerythrine and sanguinarine, induce protection of residues A4 and A5. This protection pattern is not observed for any of the other ligands tested and does not correlate with the opposed gene expression response of the riboswitch between chelerythrine and sanguinarine (activating and nonactivating, respectively). DMS reacts with N1 of both adenine and cytidine; these nucleotides compose over 70% of the four-way junction of the aptamer domain (Fig. 1*C*). Unexpectedly, increased DMS reactivity was only observed for one of the ligands tested, staurosporine, at a single residue, A5 (Fig. S3*H*). Overall, the nucleotide reactivities suggest the ligands are bound by the J1/2 element and that this adenine-rich sequence element forms a promiscuous binding pocket.

### Activating ligands impart tRNA-like structure to *yjdF*

To examine whether, from the point of view of the ribosome, the *yjdF* aptamer is tRNA like, we employed a ribosome filter binding assay (18, 19). Azaaromatic compounds do not substantially alter the affinity of tRNA^Lys^ for empty *Thermus thermophilus* 70S ribosomes (Fig. S4*A*). We next measured binding of the *S. aureus yjdF* wt aptamer domain and TLC mutant (Fig. S4, *C*–*E*). The *apo*-*yjdF* and *apo*-*yjdF* TLC exhibited only modest affinity for ribosomes (apparent *K*_*d*_ >100 nM). The wt *yjdF* and the TLC mutant also exhibited modest affinity for ribosomes in the presence of the nonactivating ligands sanguinarine or lumichrome (apparent *K*_*d*_ >100 nM). These two RNAs, however, exhibited substantial affinity for ribosomes when bound to the activators chelerythrine (*K*_*d*_ = 6 ± 3 nM and 5 ± 3 nM, respectively; mean ± SD, *n* = 3) or harmine (*K*_*d*_ = 14 ± 2 nM and 9 ± 2 nM, respectively).

The *S. aureus yjdF* riboswitch aptamer has acceptor and anticodon stem lengths that match the canonical (20) tRNA stems of 8 and 5 bp, respectively. The *yjdF* from *S. ratti*, with acceptor stem and anticodon stem lengths of 4 and 5 bp, respectively, has lower affinity for ribosomes than *S. aureus yjdF* (*K*_*d*_ not determined) but exhibits a similar dependence on azaaromatic compounds (Fig. S4*E*). Thus, ribosome binding by the *yjdF* riboswitch aptamer appears to be dependent on formation of a tRNA-like structure, and this tRNA-like structure is stabilized by activating ligands.

**Figure 3:**
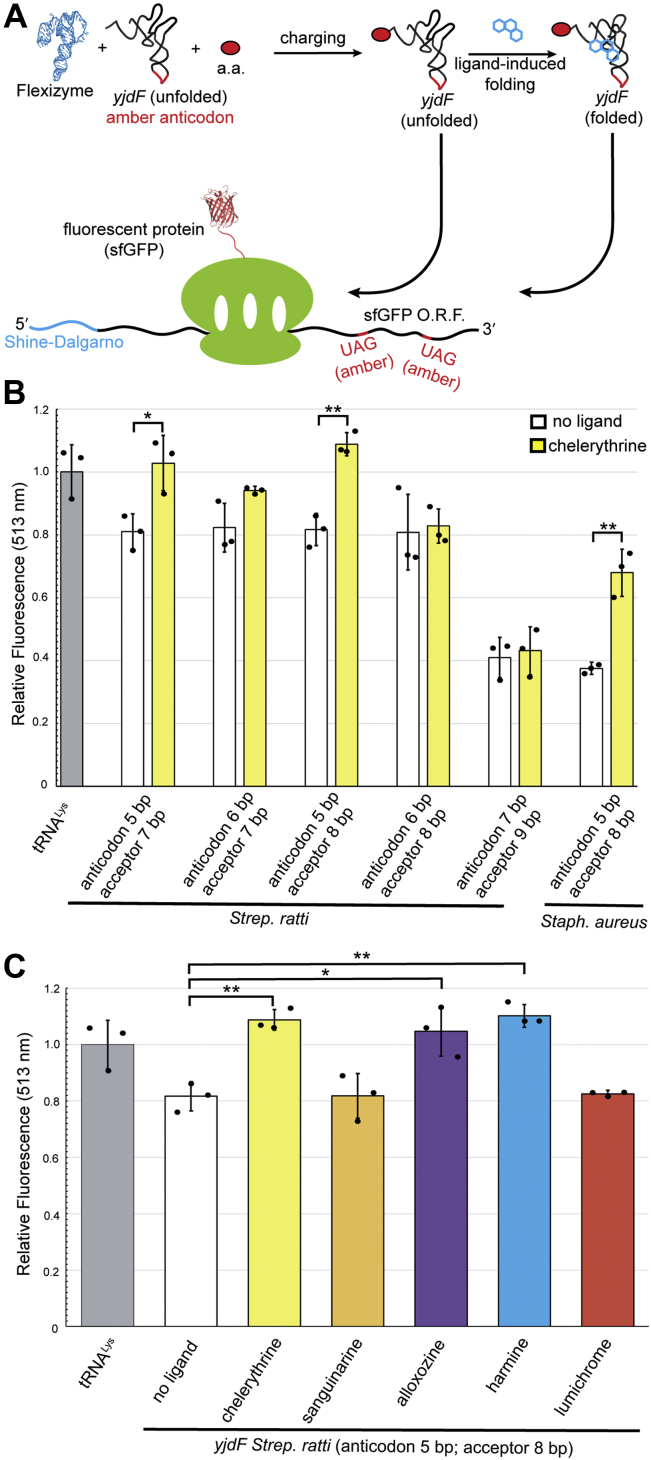
**Engineering *yjdF* into a substrate for translation elongation.***A*, experimental design of *in vitro* translation with *yjdF* variants as ribosome substrate. Briefly, tRNA and *yjdF* variants with an engineered amber (UAG) anticodon were charged with glycine. Charged RNAs were then tested for *in vitro* protein synthesis activity by mutating two glycine codons within the ORF of sfGFP to amber stop codons. Fluorescence emission was used to monitor protein yield. *B*, protein yields normalized to tRNA^Lys^_UAG_ for *yjdF* mutants with varying anticodon and acceptor stem lengths. *C*, protein yields with the *S. ratti* (anticodon 5 bp; acceptor 8 bp) substrate bound to different ligands. SAXS, small-angle X-ray scattering; SEC, size-exclusion chromatography; sfGFP, superfolder GFP; TLC, T-loop chimera.

As an independent test of the hypothesis that activating azaaromatic compounds impart a tRNA-like structure to the *yjdF* aptamer domain, we developed an assay that tests the ability of the aptamer domain to support protein synthesis as a tRNA analog (Fig. 3*A*). The *yjdF* aptamer domain was modified to include a canonical 7 nt loop with an amber anticondon (AUC) in L3, along with a 3′ ACCA acceptor tail. Using the Flexizyme *in vitro* aminoacylation system (21, 22), tRNA and *yjdF* variants were aminoacylated with glycine. We found that charged tRNA-like *yjdF*-derived constructs support ribosome-catalyzed protein synthesis of the fluorescent protein superfolder GFP, encoded by an ORF containing two glycine codons mutated to amber (UAG). Translation was most efficient in the presence of chelerythrine, and when the anticodon stem and acceptor stem lengths conformed to the canonical 5 and 8 bp lengths (Fig. 3*B*). Protein synthesis was most efficient with the activating ligands chelerythrine and harmine, whereas translation with the nonactivating ligands sanguinarine and lumichrome was indistinguishable from background (Fig. 3*C*). These results indicate that activating ligands induce a functionally tRNA-like structure of the *yjdF* aptamer domain.

### tRNA-like mRNA leaders increase protein expression

**Figure 4:**
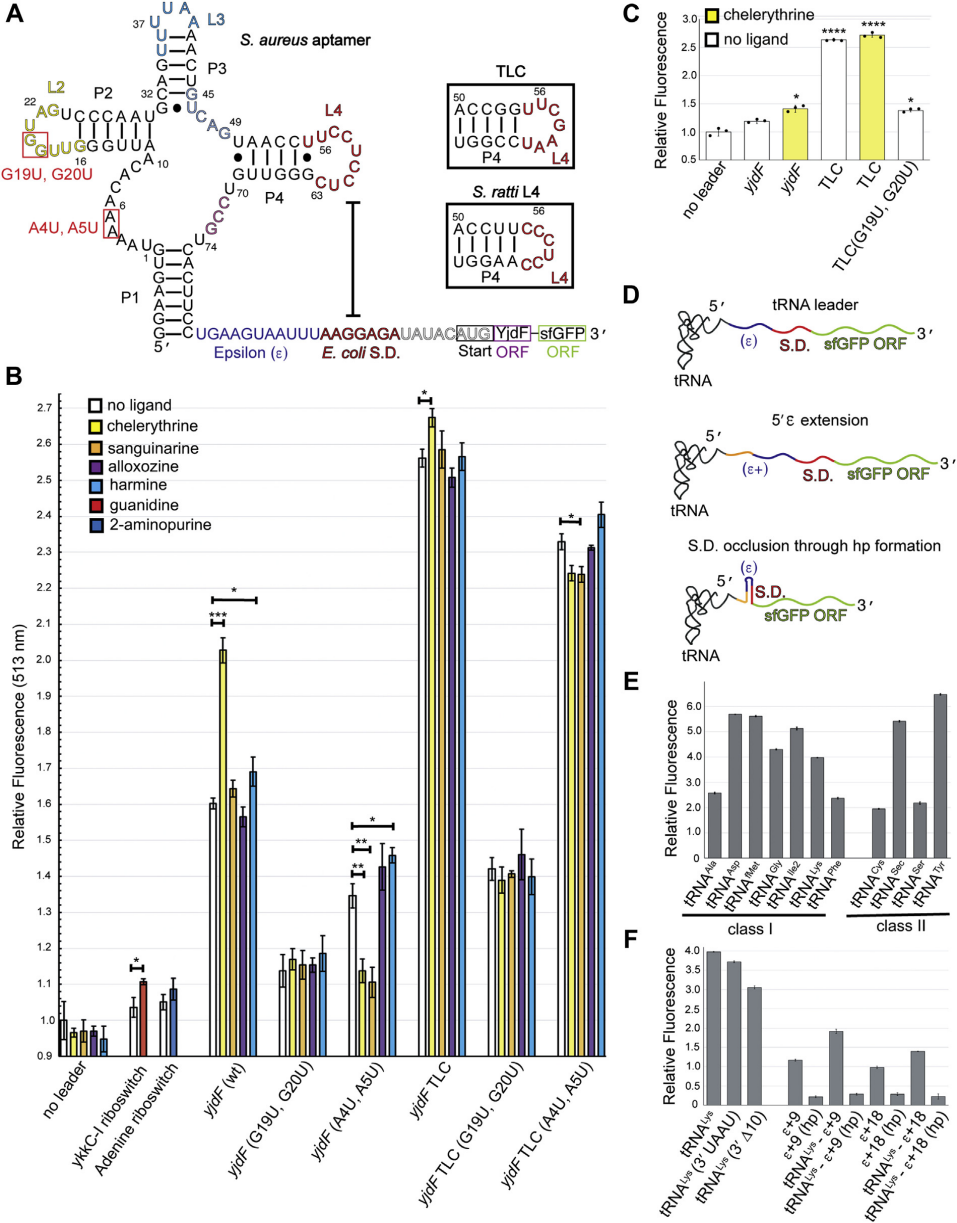
***In vitro* translation with *yjdF* and tRNA leaders.***In vitro* translation assays using the PURExpress *in vitro* translation kit. *A*, schematic of the RNA templates used to test structural importance of the *yjdF* aptamer on translation. Point mutations are labeled *red*. Chimeric TLC mutant, *boxed*. Sequence and secondary structure predictions for the TLC and *Streptococcus ratti* P4 and L4 are shown within insets. Sequence coloring same as for Figure 1, with the exception of the additional *Escherichia coli* (ε) and Shine–Dalgarno sequence. *B*, fluorescence emission relative to aptamer-free (no leader) construct using a DNA template addition to translation reaction. *C*, fluorescence emission relative to aptamer-free (no leader) construct using an RNA template added to translation reaction. *D*, schematic showing translation experiments with tRNA-leader templates. Experiments vary the tRNA identity (*top*), length of ε sequence (*middle*), and exposure of Shine–Dalgarno through occlusion (*bottom*). *E*, fluorescence emission, relative to the leaderless construct, for genes with various tRNA leaders encoded 5′ to the ε sequence. *F*, fluorescence emission, relative to the leaderless construct, for genes with tRNA^Lys^ leader. TLC, T-loop chimera.

In the off state of the *yjdF* riboswitch, between 9 and 11 nucleotides of P4 base pair with the ribosome-binding site (RBS). We generated mRNAs in which a *yjdF* aptamer domain from *S. aureus* or *S. ratti.* is located upstream of the *Escherichia coli* epsilon enhancer element and Shine–Dalgarno sequences*.* This design reduces the L4-RBS complementarity to 5 bp, thus attenuating the off state (Fig. 4*A*). We employed these derepressed mRNAs to examine whether the structure of the aptamer domain affects translation. In the absence of an aptamer domain, the expression of superfolder GFP was unaltered by azaaromatic compounds (Fig. 4*B*). Relative to this basal expression level, the aptamer domains of the *ykkC*-I riboswitch (23, 24) and *add* adenine riboswitch (25, 26), both lacking complementarity to the RBS sequence, only marginally enhanced expression, even in the presence of their cognate ligands (Fig. 4*B*). Notably, in this mRNA design, the *yjdF* aptamer domain enhanced translation by 60% (relative to the aptamer-free mRNA). Addition of the activators chelerythrine and harmine further increased translation by 40% and 9%, respectively, whereas sanguinarine and alloxozine imparted no further increase of expression. A double point mutation in L2 (G19U, G20U) reduced translation to near background; this mutant also lost ligand responsiveness. G19 and G20 are conserved in >97% of available *yjdF* riboswitch sequences (3), and their counterparts in tRNA make essential long-range tertiary contacts in the elbow region (27, 28). The binding-pocket double mutant (A4U, A5U) reduced the overall protein yield. However, the ligands chelerythrine and sanguinarine, which induced protection of these residues in our SHAPE assay, become inhibitors of protein synthesis. Alloxozine and harmine had little impact on translation of this mRNA.

tRNA genes often contain internal transcription promoters responsible for increased expression (29). To test if *yjdF* mutants are reducing protein expression through lower transcription, we also employed purified mRNA (rather than coupled transcription–translation) in our *in vitro* translation assays (Fig. 4*C*) and found that relative expression levels were similar regardless of whether template DNA or mRNA was used as input to the assay. Together, these data suggest that not only the exposure of the Shine–Dalgarno sequence but also the structure of the *yjdF* aptamer domain itself enhances protein expression.

To examine if tRNA-like elements present in *cis* to an ORF generally modulate translation, we prepared mRNAs that incorporate tRNA-like structures upstream of the Shine–Dalgarno sequence (Fig. 4*D*). These *cis*-tRNA elements increased translation between 2-fold and 6.3-fold relative to the leaderless construct (Fig. 4*E*). Expression levels did not correlate with the length of the variable loop of the *cis*-tRNA or the sequences of the D-loop and T-loop. Thus, *cis-*tRNA^Lys^ and *cis-*tRNA^Phe^ have identical D-loop and T-loop sequences but differed by approximately twofold in expression level. To test the impact of elongation factor thermo unstable on the translation of *cis-*tRNA-containing mRNA, we mutated the 3′ ACCA acceptor of the *cis*-tRNA to UAAU (which minimizes elongation factor thermo unstable affinity to authentic tRNA (30)) and found that the mutation leads to minimal loss of expression (Fig. 4*F*). Complete removal of the 3′ ACCA and the acceptor stem reduced expression by 25%. These data imply that it is the tertiary structure of the *cis*-tRNA elements, including *yjdF* riboswitch aptamer domains stabilized through binding of activating azaaromatics, which enhances translation.

## Discussion

A remarkable feature of the *yjdF* riboswitch is that it can bind diverse and chemically distinct ligands, yet it can make opposite gene regulatory decisions upon binding nearly identical ligands. Our analysis reveals that this riboswitch aptamer domain adopts a tRNA-like fold when bound to activating ligands; indeed, sequence conservation indicates that the *yjdF* riboswitch is related to tRNA^Lys^ through either homology or mimicry. Chemical probing and mutagenesis indicate that the ligand-binding site of the riboswitch is within the non–tRNA-like purine-rich J1/2 junction, and that binding of activating ligands stabilizes a tertiary interaction between loops L2 and L4. Though structurally homologous to tRNA, the riboswitch has evolved to require activating ligands to stabilize the canonical L-like fold. Upon activator ligand binding, the structure of the *yjdF* riboswitch so resembles tRNA that, when engineered to contain a CCA terminus and aminoacylated, its isolated aptamer domain can support translation. Since the pattern of nucleotide reactivity within J1/2 is not descriptive of the expression response induced by these ligands, the atomistic details of molecular recognition by the riboswitch will have to await high-resolution structures. Nonetheless, our work demonstrates that the tRNA fold can be repurposed to respond to, and discriminate between, nearly identical ligands.

In addition to its universal function in protein synthesis, tRNAs have acquired numerous roles ranging from substrates in cell wall biosynthesis to activators of stress signaling pathways (31, 32, 33, 34, 35, 36, 37). Moreover, the centrality of tRNA in translation has resulted in unrelated molecules adopting tRNA-like folds (19, 38, 39). While our work demonstrates that the tRNA-like fold of *yjdF* supports a functional ligand-responsive genetic switch, our analyses also suggest that the tRNA-like fold of the activator-bound aptamer domain enhances translation through direct interaction with the ribosome. Since the aptamer domain of the riboswitch cannot be aminoacylated (as it has no free 3′ terminus), the *yjdF* riboswitch aptamer domain is unlikely to occupy the A-site or P-site. A possible mechanism of action of the riboswitch could be analogous to those of proteins such as EttA, which modulates translation (40) by binding to the ribosomal E-site. Such a mechanism would also be consistent with our serendipitous observation that some canonical tRNAs, when inserted in *cis* to the Shine–Dalgarno element of synthetic mRNA can upregulate translation in a constitutive ligand-independent manner.

Initially thought to function exclusively through ligand-induced alternative folding, riboswitches employ diverse mechanisms including transacting regulation (41), mRNA degradation (42), internal transesterification (43), and chaperone-assisted folding (44). The direct binding of the tRNA-like *yjdF* aptamer domain to intact ribosomes now reveals another mechanism of gene activation by riboswitches (and potentially other noncoding RNAs). Many enzymes responsible for post-transcriptional modification of tRNA also modify tRNA mimics (45, 46). While the *in vivo* post-transcriptional modification status of *yjdF* riboswitch aptamer domain is still unknown, it is suggestive that there are instances of acetyl transferases and dihydrouridine synthetases under control of *yjdF* riboswitches (3, 4). In addition, greater than 5% of the human genome encodes tRNA^Lys^ paralogs in the form of ALU elements (47); elusive human riboswitches might exist in this sequence space.

## Experimental procedures

### RNA preparation

RNAs were *in vitro* transcribed as described previously (48); purified by electrophoresis on 14% polyacrylamide (19:1 acrylamide/bisacrylamide), 1× Tris/borate/EDTA, 8 M urea gels; electroeluted from gel slices; washed once with 1 M KCl; and desalted by ultrafiltration, filtered (0.1 μm cutoff, Amicon Ultrafree-MC; Millipore), and stored at −20 °C.

### SEC

To monitor oligomerization and response to ligand binding, 10 μM RNA samples were refolded in 20 mM Mops (pH 7.0), 100 mM KCl, and 10 μM EDTA in the presence or the absence of equimolar chelerythrine by heating to 95 °C for 3 min and cooling at −1 °C/min to a final temperature of 25 °C. After thermal anneal, 5 mM of MgCl_2_ was added and samples were incubated at room temperature for 10 min. Samples were run on an ÄKTApurifier at 1 ml/min over a Superdex 200 Increase size-exclusion column (GE Life Sciences) at room temperature using a running buffer composed of 20 mM Mops (pH 7.0), 100 mM KCl, 10 μM EDTA, and 5 mM MgCl_2_. Analyses containing ligand were supplemented with 60 nM chelerythrine. Absorbance was monitored at 260, 280, and 295 nm.

### SAXS

SAXS experiments were performed at beamline 12-ID-B of The Advanced Photon Source at Argonne National Laboratories. Samples were prepared and purified by SEC as described previously apart from MgCl_2_ being supplied in 2.5 mM. Sample concentration ranges from 0.25 to 0.9 mg/ml were passed through a flow cell, during which they were exposed to the X-ray beam (12 keV) for 1.0 s, followed by a 1.0 s rest time. Forty datasets were collected per sample using a Pilatus 2M detector positioned at 3.6 m from the sample capillary. Prior to averaging, each dataset was examined for radiation damage and aggregation using Igor Pro (WaveMetrics). Guinier analysis was performed using Igor Pro. Indirect Fourier transformation was performed using the ATSAS programs (49), DATGNOM and GNOM.

## SHAPE chemical probing with 2-methylnicotinic acid imidazolide

SHAPE reactions were performed using 2-methylnicotinic acid imidazolide (Sigma–Aldrich) as acylating agent. Purified RNA (0.25 μM) resuspended in buffer (20 mM MOPS–KOH, pH 7.0, 150 mM KCl, and 10 μM EDTA) was annealed by heating to 85 °C for 3 min followed by incubation at room temperature for 10 min. MgCl_2_ was added to a final concentration of 3 mM after the equilibration period. Ligands were added to a final concentration of 2.5 μM and followed by incubation at room temperature for 5 min. Reactions were carried out at 21 °C for 20 min with either 100 mM dimethyl sulfoxide (control) or 2-methylnicotinic acid imidazolide. Reactions were quenched with the addition of 90 μl of 300 mM KCl solution, 1 μl glycogen, and 300 μl of ethanol, and incubated at −80 °C for 1 h. Reverse transcription reactions were performed using SuperScript IV (Thermo Fisher Scientific) and 5′ P^32^ phosphate radiolabeled primers at 50 °C for 20 min. Remaining RNA was hydrolyzed by adding 1 M NaOH and incubated at 85 °C for 10 min. Primer extension reactions were precipitated and resuspended in denaturing loading buffer (95% formamide and 22.5 mM EDTA) with 0.1% xylene cyanol and 0.1% bromophenol blue loading dyes. Samples were resolved using 10% denaturing PAGE, exposed to phosphor image screen (GE Healthcare), and scanned with a GE Typhoon phosphor imager.

### DMS chemical probing

Purified RNA (0.25 μM) resuspended in buffer (20 mM Mops–KOH, pH 7.0, 150 mM KCl, and 10 μM EDTA) annealed by incubating at 85 °C for 3 min followed by incubation at room temperature for 10 min. MgCl_2_ was added to a final concentration of 3 mM after the equilibration period. Ligands were added to 2.5 μM followed by a 5 min incubation at room temperature. About 1% v/v ethanol (control) or DMS was added per reaction and incubated at 21 °C for 4 min. Reactions were quenched with the addition of 45 μl of β-mercaptoethanol, 45 μl 600 mM KCl solution, 1 μl glycogen, and 300 μl of ethanol, and incubated at −80 °C for 1 h. Primer extension and fractionation of complementary DNA were performed as described previously for SHAPE.

### Fluorescence measurements

Fluorescence scans were recorded on a Photon Technologies International/820 Photomultiplier Detection System set to measure at excitation wavelength of 342, 350, and 440 nm for harmine, sanguinarine, and chelerythrine, respectively. Emission was recorded from 400 to 460 nm (harmine) or 500 to 600 nm (chelerythrine or sanguinarine). Fluorescence signals were averaged over 5 s at a 2 nm bandwidth. Solutions contained 20 mM Mops (pH 7.0), 100 mM KCl, 10 μM EDTA, 5 mM MgCl_2_, and 50 nM of fluorophore. Preannealed RNA was added in matching buffer (no fluorophore) and allowed to equilibrate for 1 min prior to measurement at 20 °C.

Binding affinity measurements were performed on a Photon Technologies International/820 Photomultiplier Detection System at an excitation of 342 nm and emission of 430 nm. Solutions contained 20 mM Mops (pH 7.0), 100 mM KCl, 10 μM EDTA, 5 mM MgCl_2_, and 50 nM harmine. Fluorescence signals were averaged over 30 s for each manual titration point. Data from three independent runs were averaged and fit to the Hill equation, assuming two independent binding sites (Equation 1).

(1) $$\left(I_{\text{mid}}-\;I_{0}\right)*\;\left(\frac{(K_{1\;}*C_{\text{RNA}})ˆn1}{1+\left(K_{1\;}*\;C_{\text{RNA}}\right)ˆn1}\right)+\left(I_{f}-\;I_{\text{mid}}\right)*\;\left(\frac{(K_{2\;}*C_{\text{RNA}})ˆn2}{1+\left(K_{2\;}*\;C_{\text{RNA}}\right)ˆn2}\right)$$

### Filter binding measurements

Ribosome-binding experiments were performed using empty *T. thermophilus* HB27ΔL9 ribosomes obtained from Harry Noller and Laura Lancaster (University of California, Santa Cruz). Ribosomes were diluted in 20 mM Hepes (pH 7.0), 100 mM NH_4_Cl, 5 mM MgCl_2_, and 0.5 mM spermine just prior to incubation with RNA. 5′ ^32^P-labled RNA samples were diluted in 20 mM Hepes (pH 7.0), 100 mM NH_4_Cl, 5 mM MgCl_2_, 0.5 mM spermine ± 60 nM chelerythrine or sanguinarine at 100 cpm/μl. To minimize scavenging of ligands by ribosomes that results in *yjdF* unfolding, RNA/ribosome mixtures were incubated for exactly 5 min at room temperature. Filter binding was performed on a DotBlot vacuum manifold with Nitrocellulose (Cytiva) and Hybond-N+ nylon (Amersham) membranes. Membranes were pretreated by washing with Milli-Q H_2_O for 5 min, soaked in 0.1 M NaOH for 15 min, washed with Milli-Q H_2_O, soaked in 0.1 M Hepes (pH 7.0) for 5 min, and then equilibrated in binding buffer for a minimum of 45 min. After adsorption, membranes were allowed to dry in air and then exposed to an Amersham phosphor imaging screen for approximately 12 h. Screens were imaged on an Amersham Typhoon Imager at 100 μm/pixel. Data were analyzed using Image Studio Lite (LI-COR).

### *Trans in vitro* protein synthesis

*yjdF* variants and tRNA^Lys^ were aminoacylated using Flexizyme (dFx) and the glycine–dinitrobenzyl ester substrate as previously described (50). Just prior to *in vitro* translation, aminoacylation reactions were resuspended after ethanol precipitation in 30 mM sodium acetate (pH 5.2), and concentrations were determined on a NanoDrop spectrophotometer. About 12.5 μl reactions of PURExpress ΔRF123 (NEB) protein expression kits were used to test *yjdF* activity as a protein synthesis substrate. About 3000 ng of resuspended aminoacylation reaction were added per reaction, ±1 μM of appropriate ligand. Reactions were incubated for 12 h at 30 °C. Protein synthesis yield was determined by fluorescence measurements on a Photon Technologies International/820 fluorimeter at an excitation wavelength of 498 nm and emission wavelength of 513 nm (bandwidth = 2 nm). Fluorescence signals were averaged for 30 s and reported as the mean of three independent measurements.

### *In vitro* protein synthesis with *yjdF*, tRNA, or tRNA-like leaders

Purified PCR-amplified gene was added to a final concentration of 70 ng/μl in 12.5 μl reactions of PURExpress protein expression system. The riboswitch ligands were supplied in 1 μM concentration, when noted. Reactions were incubated for 12 h at 25 °C. Protein synthesis yield was determined by fluorescence emission on a Photon Technologies International/820 fluorimeter at an excitation wavelength of 498 nm and emission wavelength of 513 nm (bandwidth = 2 nm). Fluorescence signals were averaged for 30 s and reported as the mean of three independent measurements.

### Quantification and statistical analysis

Fluorescence enhancement and emission are reported as the mean of at least three measurements with error reported by SEM. Data labeled with *asterisks* report single-factor ANOVA with significance *p* < 0.05 or *p* < 0.01.

## Data availability

All data generated are contained within the article. Data and material requests should be made to R. J. T.

## Supporting information

This article contains supporting information.

## Conflict of interest

The authors declare that they have no conflicts of interest with the contents of this article.
