# Supplementary material for: The bacterial yjdF riboswitch regulates translation through its tRNA-like fold
Source: J Biol Chem. 2022 Apr 12;298(6):101934. doi: 10.1016/j.jbc.2022.101934 (PMC9142559; doi:10.1016/j.jbc.2022.101934)
Supplement: Supplemental Figures S1–S4 and Table S1 [file mmc1.docx]

**Supplementary Material**

**The bacterial *yjdF* riboswitch regulates translation through its tRNA-like fold**

Robert J. Trachman III^1,2*^, Luiz F. M. Passalacqua^1^, and Adrian R. Ferré-D’Amaré^1^

^1^ Biochemistry and Biophysics Center, National Heart, Lung, and Blood Institute, 50 South Drive MSC 8012, Bethesda, MD 20892-8012, USA

^2^ Present Address: Nucleic Acid Replication Division, New England Biolabs, Ipswich, MA, 01938

*Correspondence: R.J.T. [rtrachman@gmail.com](mailto:rtrachman@gmail.com) or A.R.F. adrian.ferre@nih.gov

Key words: tRNA, RNA structure, riboswitch, translation

**
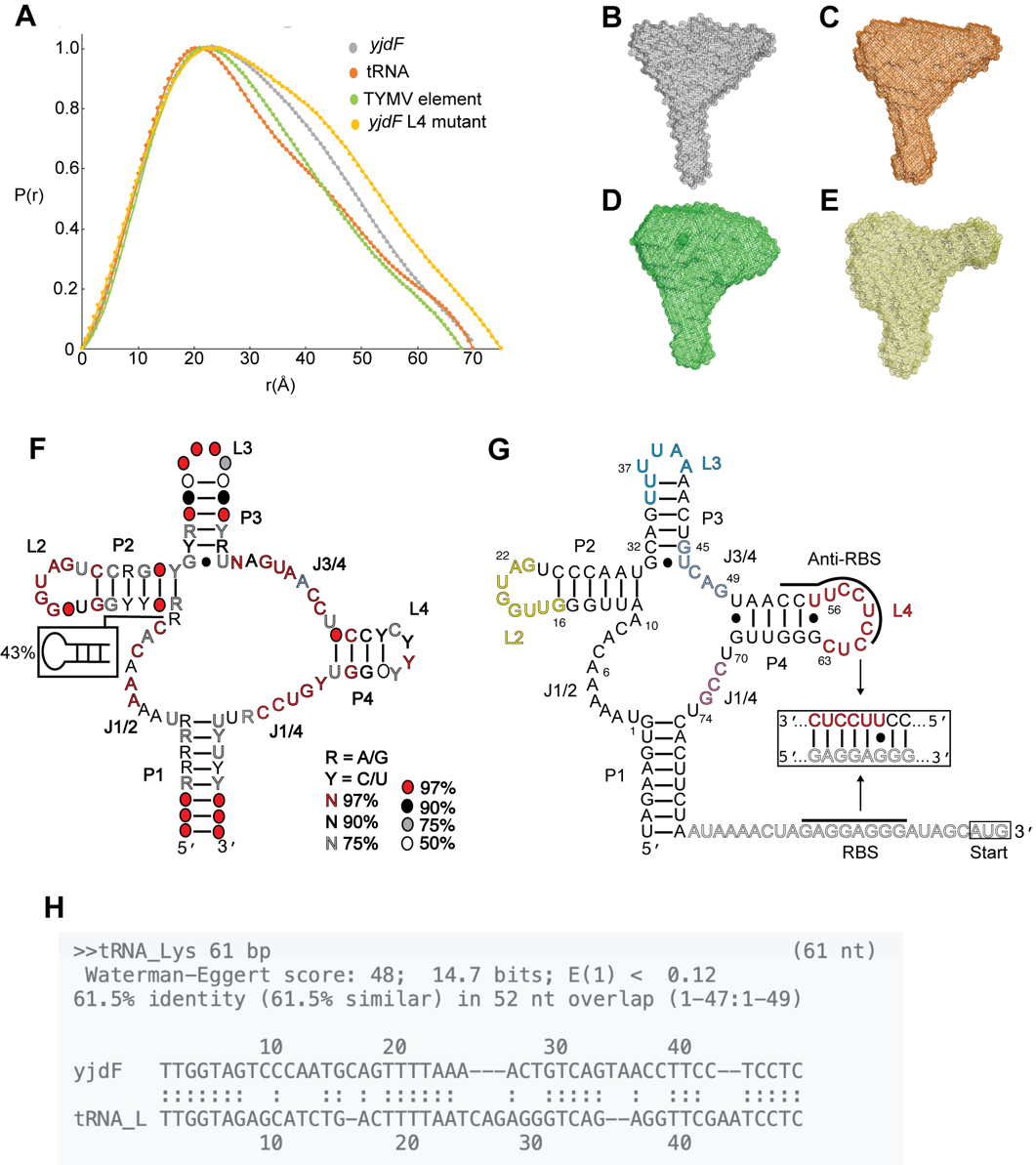
**

**Figure S1. Analysis of tRNA-like structures. (A)** Electron-pair distribution function [*p*(*r*)] plots for the tRNA-like RNAs *yjdF*, tRNA^Lys^, TYMV, and the *yjdF* L4 mutant. Envelopes calculated using real pace scattering curves in (**A)** are shown for (**B)** *yjdF*, (**C)** tRNA^Lys^, (**D)** TYMV, and (**E**) *yjdF* L4 mutant. (**F)** secondary structure prediction of the *yjdF* aptamer domain using consensus sequence and covariation (figure panel adapted from Li et al. 2016). (**G)** Secondary structure prediction of S. aureus *yjdF* aptamer domain and leader sequence using RNAfold (Hoffacker et al. 1994). Boxed inset shows predicted H-type pseudoknot formed in the off-state. (**H)** alignment of *S. aureus yjdF* aptamer residues 17-73 and *S. aureus* tRNA^Lys^ residues 15-76.

**
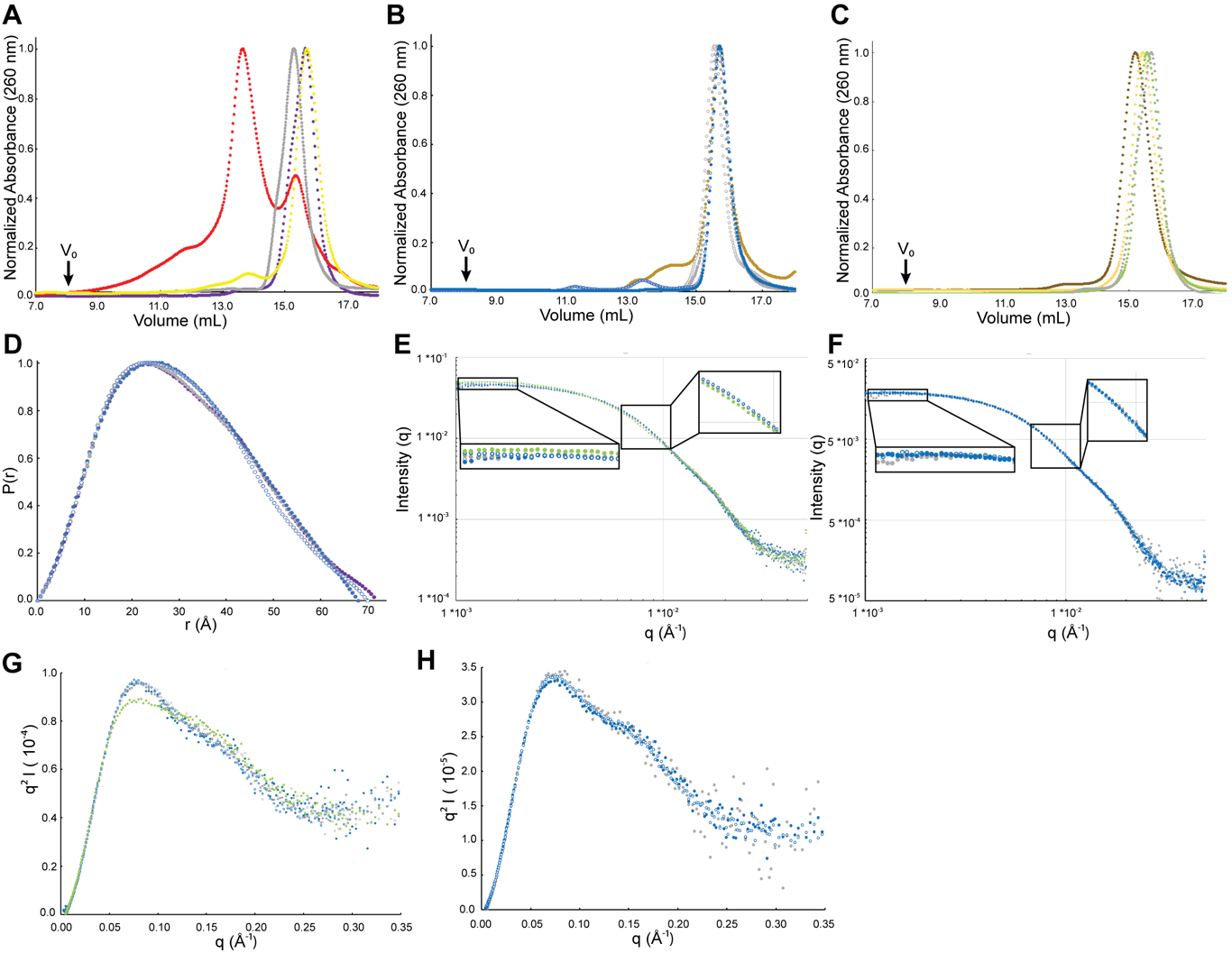
**

**Figure S2. Folding of the *yjdF* aptamer domain.** Size exclusion chromatography (SEC) and small angle X-ray scattering (SAXS) analyses of *yjdF* variants. Throughout, closed circles denote conditions in presence of 60 nM chelerythrine, open circles denote conditions in absence of ligand, grey circles (*wild type*). **(A)** SEC of *E. pallens* circular permutants ∆P2 (red), ∆P3 (purple), and ∆P4 (yellow). **(B)** SEC of *S. ratti* L4 mutants, TLC (blue), TLC+1 (honey). **(C)** SEC of *E. pallens* TYMV mutants. **(D)** P(r) plots for *S. ratti* *yjdF* mutants TLC (blue), circ∆P3 (purple). **(E)** Log-log plots for the *E. pallens yjdF* variant and mutants TLC( blue). Subpanels show magnified views of low-q and mid-q regions. **(F)** Log-log plots for the *S. aureus yjdF* variant and mutants TLC( blue). Subpanels show magnified views of low-q and mid-q regions. **G.** Kratky plot of scattering profiles from **(E)**. **(H)** Kratky plot of scattering profiles from **(F)**.

**
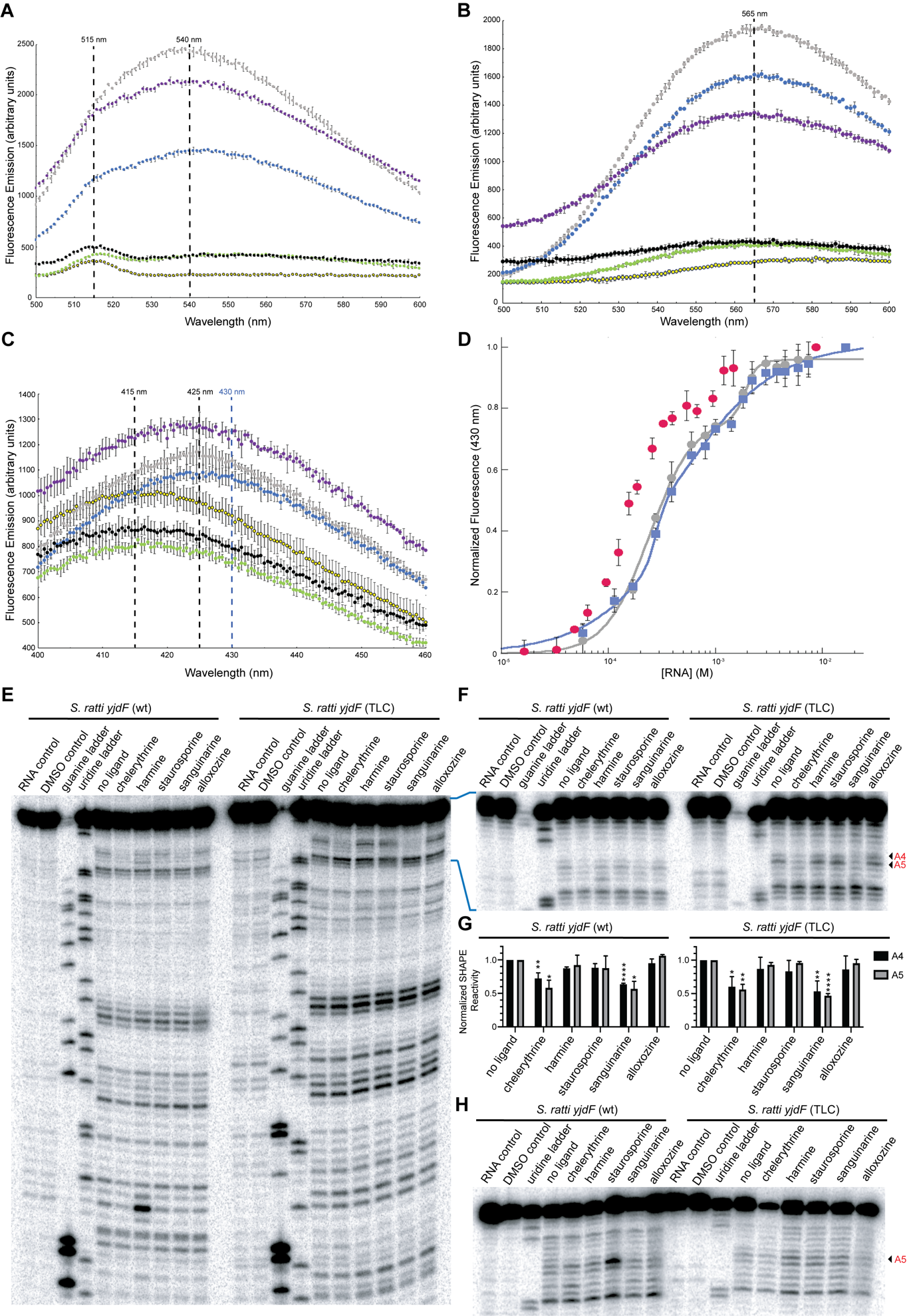
**

**Figure S3. Fluorescence emission of azaaromatic compounds.** Fluorescence emission of *S. ratti yjdF wt* (grey), *yjdF* TLC (blue), circ∆L3 (purple), tymvD/T (green), *S. aureus* tRNA^Lys^ (black) in complex **(A)** chelerythrine, **(B)** sanguinarine, or **(C)** harmine. Unbound fluorophores are plotted yellow with black outline. Black dashed lines indicate the emission maxima for either bound or unbound fluorophores. Blue dashed line indicates emission wavelength used for harmine fluorescence binding assay. Error bars represent standard error from three replicates. **(D)** Normalized fluorescence emission of harmine (430 nm) wt *S. ratti yjdF* (grey), *S. ratti yjdF* TLC (blue), and *S. ratti yjdF* circ∆P3 (red). Fits are to a two-state Hill equation. Error bars represent standard error for at least three replicates. **(E)** NIA, SHAPE reactions. (**F)** zoom in of same reactions in (**E**) with longer running time. **(G)** Normalized SHAPE reactivity for residues A4 and A5 with various azaaromatics. Error bars report standard error, asterisks indicate significance from single factor ANOVA (P < 0.01). **(H)** DMS chemical probing data with zoom-in view of binding-pocket nucleotides.

**
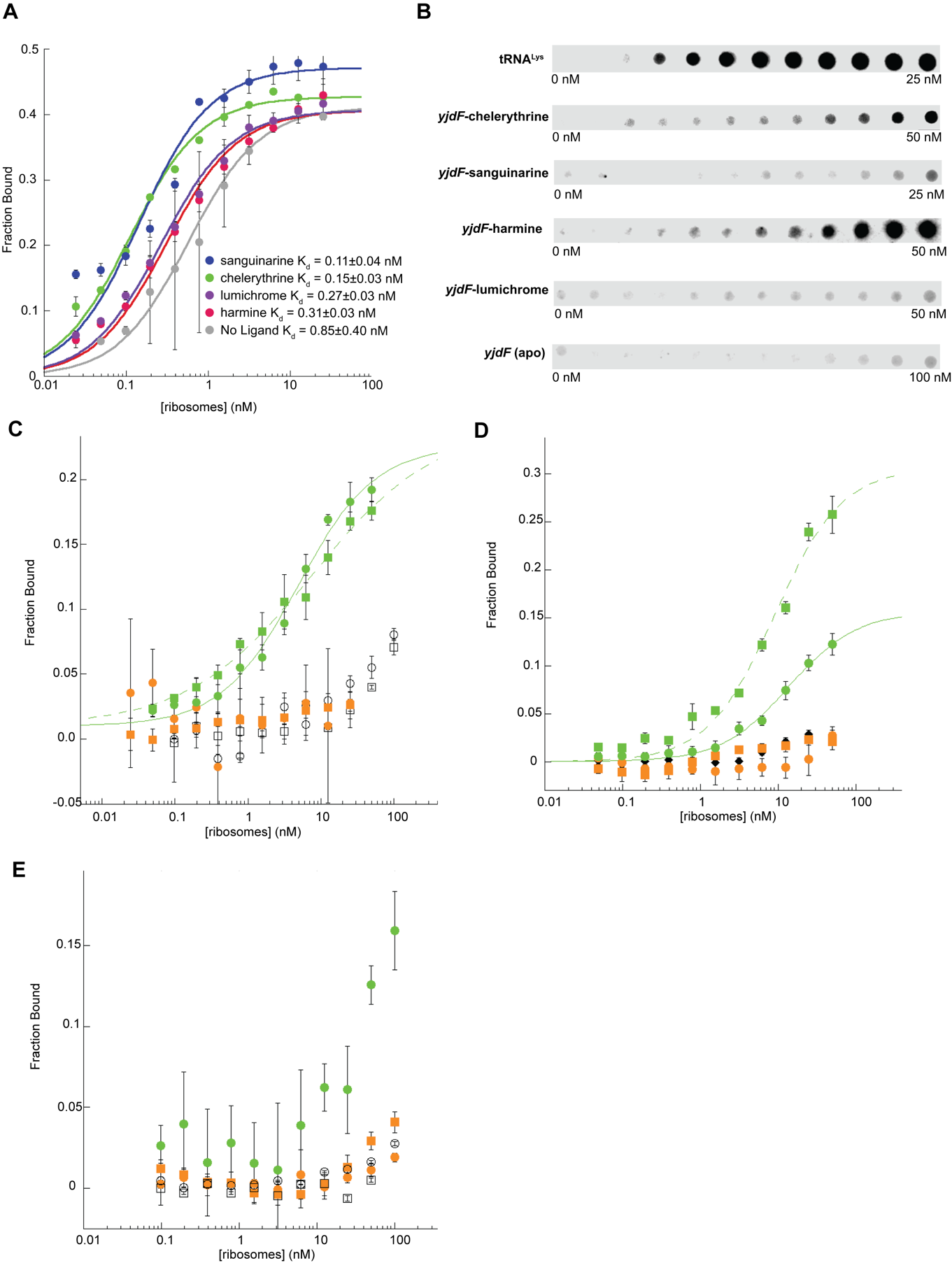
**

**Figure S4. Ribosome filter binding assay.** Filter binding data for *yjdF* and controls to Thermus thermophilus 70S ribosomes. **(A)** tRNA^Lys^ binding to empty *Thermus thermophilus* ∆L9 70S ribosomes in the presence of azaaromatic compounds. (**B)** Examples of ribosome binding titrations on nitrocellulose for tRNA^Lys^ and various *staph. aureus* *yjdF* complexes. **(C)** Ribosome binding to *S. aureus yjdF* (circles) and *S. aureus yjdF* TLC (squares). Isotherms in the absence of ligand (black), 60 nM sanguinarine (orange), and 60 nM chelerythrine (green). **(D)** Ribosome binding to *S. aureus yjdF* (circles) and *S. aureus yjdF* TLC (squares), or ykkC-I riboswitch (black). Isotherms for 2 μM lumichrome (orange), and 2 μM harmine (green). **(E)** Ribosome binding to *S. ratti* *yjdF* (circles) or *S. ratti* *yjdF* TLC (squares). Isotherms in the absence of ligand (black), 60 nM sanguinarine (orange), or 60 nM chelerythrine (green). Data are fit to the Hill equation with error bars showing standard error for at least three replicates.

**Table 1. Names and sequences of circular permutants.** Engineered loops colored in red.

| Species | Construct Name | Sequence |
| --- | --- | --- |
| Enterococcus pallens | wt | CGAGCATAAAAACACGAGGCGGTTGGTAGTCCGCCGCATGAAAATGTCAGTAGCCTTCCCTCCCGGGTCGTCCATTGCTCG |
|  | circ∆L2 | CCGCCGCATGAAAATGTCAGTAGCCTTCCCTCCCGGGTCGTCCATTGCTCGTTCGCGAGCATAAAAACACGAGGCGG |
|  | circ∆L3 | GTGTCAGTAGCCTTCCCTCCCGGGTCGTCCATTGCTCGTTCGCGAGCATAAAAACACGAGGCGGTTGGTAGTCCGCCGCAC |
|  | circ∆L4 | GGGTCGTCCATTGCTCGTTCGCGAGCATAAAAACACGAGGCGGTTGGTAGTCCGCCGCATGAAAATGTCAGTAGCCT |
| Streptococcus ratti | wt | GGTATAAAAACACATTGCAGTTGGTAGTCTGCAAGCATCATCAGATGTCAGTAACCTTCCCTCCAAGGTCGTCCCATACC |
|  | circ∆L2 | CTGCAAGCATCATCAGATGTCAGTAACCTTCCCTCCAAGGTCGTCCCATACCGTTCGCGGTATAAAAACACATTGCAG |
|  | circ∆L3 | GATGTCAGTAACCTTCCCTCCAAGGTCGTCCCATACCGTTCGCGGTATAAAAACACATTGCAGTTGGTAGTCTGCAAGCATC |
|  | circ∆L4 | AAGGTCGTCCCATACCGTTCGCGGTATAAAAACACATTGCAGTTGGTAGTCTGCAAGCATCATCAGATGTCAGTAACCTT |
| Clostridium difficile | wt | TAAAGATAAAAACACAGTCTGGTTGGTAGTCCAGACGCAGCAATAGCTGTCAGTAACCTGCCTCCTTGGTTGTCCGTTCTTTA |
|  | circ∆L2 | CCAGACGCAGCAATAGCTGTCAGTAACCTGCCTCCTTGGTTGTCCGTTCTTTATTCGTAAAGATAAAAACACAGTCTGG |
|  | circ∆L3 | GCTGTCAGTAACCTGCCTCCTTGGTTGTCCGTTCTTTATTCGTAAAGATAAAAACACAGTCTGGTTGGTAGTCCAGACGCAGC |
|  | circ∆L4 | GGTTGTCCGTTCTTTATTCGTAAAGATAAAAACACAGTCTGGTTGGTAGTCCAGACGCAGCAATAGCTGTCAGTAACC |
